# A Mysterious Paratracheal Mass: Parathyroid Carcinoma

**DOI:** 10.7759/cureus.9126

**Published:** 2020-07-11

**Authors:** Qian Zhang, Khine S Shan, Neethu Gopisetti, Thomas Yoon, Iqra Iqbal

**Affiliations:** 1 Internal Medicine, Abington Hospital - Jefferson Health, Abington, USA; 2 Internal Medicine, University of Maryland Medical Center, Baltimore, USA; 3 Internal Medicine, Abington Memorial Hospital, Abington, USA

**Keywords:** parathyroid carcinoma, acute hypercalcemia, hypercalcemia, parathyroid cancer, pth

## Abstract

Parathyroid carcinoma is a rare cause of primary hyperparathyroidism. We detail a 60-years-old gentleman who was otherwise healthy presented to the hospital due to acute encephalopathy. He was subsequently found to have parathyroid carcinoma as the cause of the acute encephalopathy with impressive serum calcium and parathyroid hormone levels. The parathyroid carcinoma was later surgically resected with the diagnosis confirmed via pathology specimen. The patient was safely discharged from the hospital with recommendations of close routine outpatient followup.

## Introduction

Parathyroid carcinoma is a rare cause of primary hyperparathyroidism [1]. It is accountable for less than one percent of all sporadic primary hyperparathyroidism [2]. According to a systemic review conducted from 1995-2003 by Ruda et al., parathyroid carcinoma accounted for 0.74% of cases out of the total study population of 22,225 patients [3]. The diagnosis of parathyroid carcinoma is often made status post resection of the tumor. It is important to recognize parathyroid carcinoma in the early phases as a prompt aggressive surgical approach is essential for the treatment of the disease [2]. 

## Case presentation

The patient was a 60-years-old gentleman with no pertinent past medical history presented to the emergency department (ED) due to altered mentation. He was found to be confused, acting strangely in his home by his neighbor that prompted the urgent ED visit. Initial laboratory findings: temperature 98.1° F, blood pressure 149/81 mmHg, respiratory rate 26 breaths per minute, heart rate 100 beats per minute, and oxygen saturation 94% on room air. The patient was alert and oriented to self and unable to follow verbal commands. Laboratory findings showed a negative urine drug screen, white blood cells of 18.3 K/uL with neutrophilic predominance along with lactic acid of 3.2 mEq/L, and negative severe acute respiratory syndrome coronavirus 2 (SARS-CoV-2). Arterial blood gas finding was otherwise unremarkable. The initial serum calcium was found to be 21.5 mg/dL with parathyroid hormone (PTH) of 1615.1 pg/mL. The endocrinologist recommended intravenous fluids, calcitonin, cinacalcet, and pamidronate for hypercalcemia treatment. A computerized tomography (CT) scan of the head was negative for acute intracranial abnormalities. The chest X-ray was unremarkable for inflammatory processes. There were concerns of sepsis given acute encephalopathy along with lactic acidosis despite otherwise being hemodynamically stable. He was subsequently treated empirically with vancomycin, ceftriaxone, ampicillin, obtained cultures, and admitted into the medical intensive care unit (MICU) for further evaluation. He remained stable overnight. 

On the second day of hospitalization, the patient remained disoriented, confused, and restless in the setting of hemodynamic stability. The differential diagnosis included meningoencephalitis as nuchal rigidity was found on physical examination. Lumbar puncture and magnetic resonance imaging of the brain was not performed as the patient was agitated. Dexamethasone was added to the treatment regimen. The patient remained in the MICU overnight.

On the third day of hospitalization, he became more agitated on top of confusion and restlessness. The empirical antibiotics were discontinued as there was a low clinical suspicion of an infectious process given normalization of lactic acid and negative culture data. Serum calcium level had improved from 21.5 mg/dL to 13.1 mg/dL after receiving the hypercalcemia treatment. PTH continued to trend upwards from 1615.1 pg/mL to 2419.4 pg/mL. There was a strong clinical suspicion that the acute encephalopathy was related to metabolic derangement due to severe hypercalcemia. He remained hemodynamically stable overnight. 

**Figure 1:**
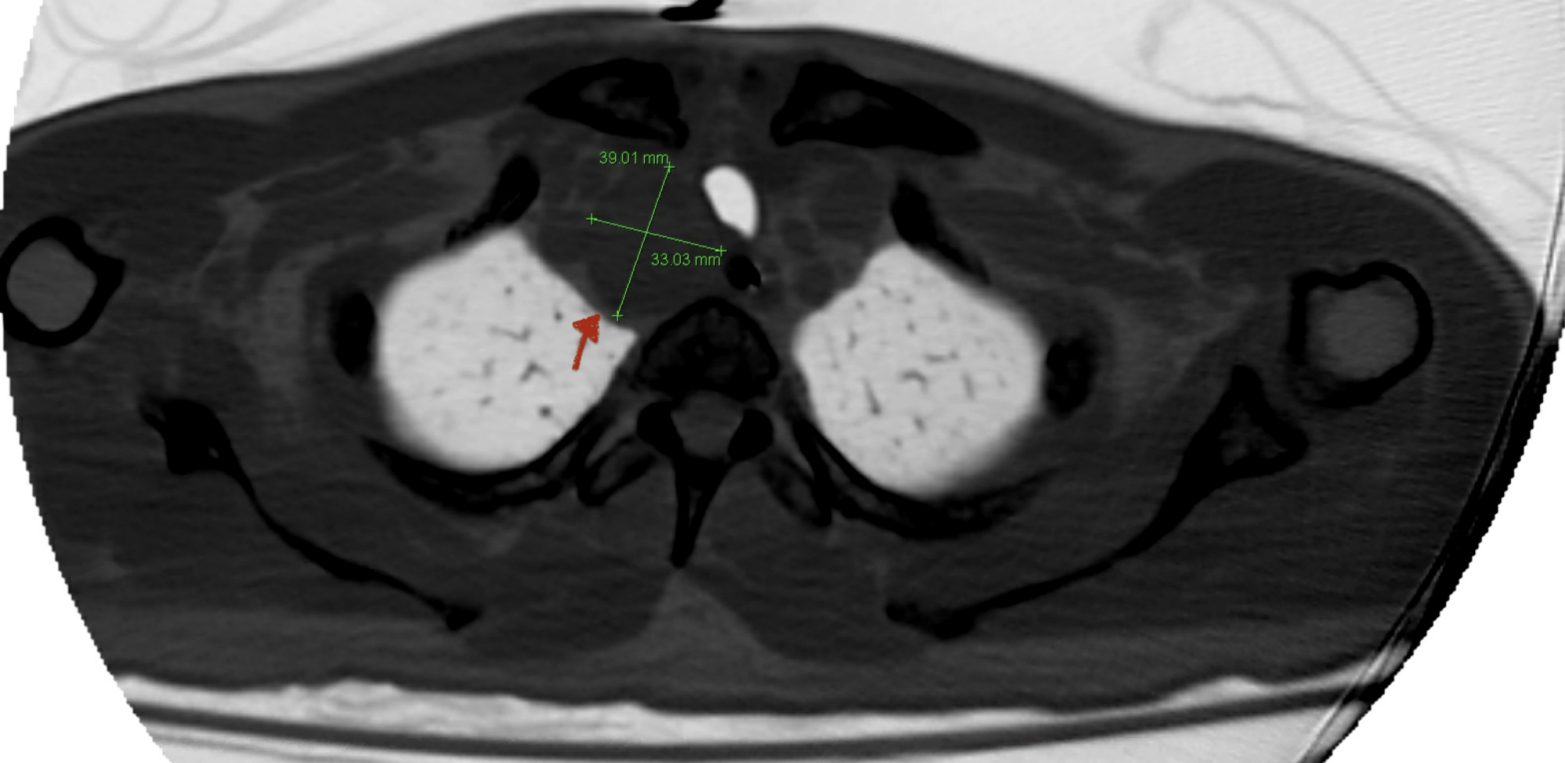
Chest CT scan A 39x33 mm mass located at the right retroclavicular location that indented and deviated the trachea to the left side.

On days 4-10 of hospitalization, the patient became more lucid as serum calcium level had normalized to 9.1 mg/dL with treatment. CT of the chest, abdomen, and pelvis was ordered as there was a strong concern for underlying malignancy given the severe hypercalcemia with elevated PTH. CT discovered a 39x33 mm mass located at the right retroclavicular location that indented and deviated the trachea to the left side (Figure 1). No other abnormal masses were discovered in the rest of the imaging study. The paratracheal mass was subsequently surgically resected and sent for pathology evaluation. 

On day 11 of hospitalization, the patient recovered well after the surgery with stable hemodynamics, adequate pain control without surgical complications. His mentation had completely returned to normal as he was alert and oriented. Postoperative serum calcium level was 7.4 mg/dL with PTH of 277.2 pg/mL. Serum calcium levels were trended for the next couple of days to ensure there were no signs of hungry bone syndrome. He was started on calcium carbonate 1250 mg daily. The patient remained to have normal serum calcium levels with elevated PTH levels for the next few days. There were no physical examination findings of paresthesias or perioral numbness. Surgical pathology findings revealed parathyroid carcinoma. He was subsequently discharged with 500 mg of calcium tablets twice a day. He was recommended to have close follow up in the outpatient setting for the continuation of care. 

## Discussion

Acutely altered mentation is one of the common reasons that require hospitalization. An acute cognitive impairment or delirium is often associated with an underlying illness [4]. There are multiple etiologies that may contribute to acute delirium. In a prospective study conducted in 229 elderly patients by Francis et al., it was discovered that common risk factors such as abnormal electrolytes, dementia, fever or hypothermia, medication side effects, and azotemia were associated with delirium [5]. Our patient, an otherwise healthy individual with no pertinent medical history, was suddenly found to be delirious by his neighbor. He had received extensive workup in the medical intensive care unit to rule out potential causes that may explain his acute encephalopathy. There were initial concerns that his acute delirium could be contributed by an infectious process as he was treated with empirical broad-spectrum antibiotics. Later on, dexamethasone was added to his treatment regimen as there was a positive neurological finding of nuchal rigidity to cover for acute meningoencephalitis. However, these differential diagnoses were soon eliminated, as hypercalcemia was the cause of the delirium. 

Primary hyperparathyroidism is mostly caused by a solitary benign adenoma (from 80 to 85%). Nevertheless, other causes include double adenomata (from 2 to 5%), diffuse or nodular hyperplasia (from 10 to 15%), or parathyroid carcinoma (<1 %) [6]. Severe hypercalcemia is defined as serum calcium > 14 mg/dL. It is often associated with severe clinical symptoms, including stupor, confusion, lethargy, and coma [7]. The first step in managing hypercalcemia is to confirm the result of hypercalcemia via total calcium corrected for albumin. The degree of hypercalcemia could serve as an indicator of the possible underlying etiology once the value is confirmed. Serum calcium of greater than 13.5 mg/dL is unusual for hyperparathyroidism, whereas it is a strong suspicion for underlying malignancy [8]. 

Parathyroid carcinoma's important clinical manifestations are related to various factors such as the age, serum calcium level, and the parathyroid hormone level. According to data obtained from a retrospective study conducted by Quinn et al. at a tertiary referral center that investigated 3,643 patients who were referred for surgical evaluation, 18 patients were later found to have papillary carcinoma [9]. Parathyroid carcinoma patients had larger tumor sizes, higher mean serum calcium and PTH levels, and also had a higher incidence for hypercalcemic crisis comparing to patients with atypical adenomas. Furthermore, the mean serum calcium and PTH levels were 13.0 mg/dL and 489 pg/mL, respectively [9]. Interestingly, our patient had an impressive initial serum calcium level of 21.5 mg/dL along with PTH level that peaked at 2419.4 pg/mL.

We opted to perform a CT scan of the chest, abdomen, and pelvis as there was a strong suspicion of malignancy in order to evaluate for anatomical staging as well as pre-operative planning. Based on findings from a retrospective review of 733 patients with parathyroid carcinoma from the National Cancer Database from 1985 to 2006 by Asare et al., it was discovered that the age of the patients, tumor size, and sex had an impact on the five and 10 years overall survival rate of the patients [10]. The primary treatment for parathyroid carcinoma is surgical resection, as it offers the greatest chance for potential cure [11]. However, it is advised that the serum calcium levels should be controlled prior to surgery as our patient's hypercalcemia was normalized after being treated with intravenous fluids, calcitonin, cinacalcet, and pamidronate. Moreover, adjuvant radiation therapy did not show an absolute survival benefit, whereas other forms of treatment, including chemotherapy and bio-modifying agents, currently remain unproven and experimental. Unfortunately, re-operative surgery should be considered if there is a reoccurrence of malignancy or persistent carcinoma induced hypercalcemia [11]. It was noted that 33% of the patients were cured after the initial surgical resection, 33% of the patients had a reoccurrence that required reoperation, and 33% of the patients experienced a short and aggressive clinical course [12]. 

## Conclusions

Parathyroid carcinoma is a rare but potentially fatal cause of primary hyperparathyroidism. It is crucial to have prompt surgical evaluation after initial medical stabilization of the patient that often requires intensive care unit level of care. Close follow-up is essential as patients could develop complications or reoccurrence of the malignancy after the initial surgical resection. 
